# Low-Carbohydrate Diets in the Management of Obesity and Type 2 Diabetes: A Review from Clinicians Using the Approach in Practice

**DOI:** 10.3390/ijerph17072557

**Published:** 2020-04-08

**Authors:** Tara Kelly, David Unwin, Francis Finucane

**Affiliations:** 1 HRB Clinical Research Facility, National University of Ireland, H91YR71 Galway, Ireland; tarakellyrd@gmail.com; 2 The Norwood Surgery, Southport PR9 7EG, UK; unwin5@btinternet.com; 3 Bariatric Medicine Service, Centre for Diabetes, Endocrinology and Metabolism, Galway University Hospitals, H91YR71 Galway, Ireland

**Keywords:** low-carbohydrate diets, diabetes remission, lifestyle modification, obesity treatment, type 2 diabetes

## Abstract

Low-carbohydrate diets are increasingly used to help patients with obesity and type 2 diabetes. We sought to provide an overview of the evidence for this treatment approach, considering the epidemiology and pathophysiology of obesity and diabetes in terms of carbohydrate excess. We describe the mechanistic basis for the clinical benefits associated with nutritional ketosis and identify areas of practice where the evidence base could be improved. We summarize the key principles which inform our approach to treating patients with low-carbohydrate diets. The scientific controversy relating to these diets is real but is consistent with the known challenges of any dietary interventions and also the limitations of nutritional epidemiology. Secondly, notwithstanding any controversy, international guidelines now recognize the validity and endorse the use of these diets as a therapeutic nutritional approach, in appropriate patients. Thirdly, we have found that early de-prescription of diabetes medications is essential, in particular insulin, sulphonylureas, and sodium-glucose cotransporter (SGLT2) inhibitors. Fourthly, we encourage patients to eat ad libitum to satiety, rather than calorie counting per se. Furthermore, we monitor cardiovascular risk factors frequently, as with all patients with obesity or diabetes, but we do not necessarily consider an increase in low-density lipoprotein (LDL)-cholesterol as an absolute indication to stop these diets, as this is usually related to large LDL particles, which are not associated with increased cardiovascular risk. In the absence of large randomized controlled trials with cardiovascular and other hard endpoints, adopting a low-carbohydrate diet is a legitimate and potentially effective treatment option for patients with diabetes or obesity.

## 1. Introduction

With the rising prevalence of obesity and diabetes [1,2], the need to develop effective treatment options for affected individuals continues to increase. Several studies have confirmed the benefits of structured lifestyle interventions in different patient subgroups, including those with non-diabetic hyperglycemia [3], prevalent cardiovascular disease [4], or established type 2 diabetes [5,6]. However, meaningful, sustained reductions in weight over time are difficult to achieve with lifestyle modification alone [7]. In a large general practice-based cohort study of severely obese adults in the UK, the annual probability of achieving 5% weight loss was one in eight for men and one in seven for women [8]. Some suggest a meaningful improvement in health requires a weight loss of 10% [6].

Meal replacement programs can achieve typical initial weight loss of this magnitude and improve glycemic control [5,9] but weight regain can limit their longer-term efficacy [7,10]. Though usually well tolerated, these may have side effects, including constipation, dizziness, alopecia, headache, and cholelithiasis [11]. While participant retention in some meal replacement studies is good, attrition rates of 50% have been seen [7,12]. Furthermore, commercial meal replacement programs can be expensive, with some analyses suggesting they are prohibitively cost ineffective [13]. Drug treatments are available [14], but as with lifestyle interventions, there is heterogeneity both in how much weight is lost and the improvements seen in glycemic control with different medications [15]. Bariatric surgery is efficacious and cost effective [16], particularly in patients with type 2 diabetes, where sustained remission is feasible for a substantial proportion of patients [17]. However, only 10% of eligible patients would choose this option [18].

Low-carbohydrate diets have recently been advocated by some clinicians and professional societies as a valid and effective therapeutic option for diabetes and obesity [19]. However, this is a scientific area that is rife with controversy and conflicting findings which have polarised expert opinion and can cause confusion for health care professionals and their patients. In fact treating obesity and diabetes in this way is not new: Over 200 years ago, the Scottish physician John Rollo described resolution of glycosuria with carbohydrate restriction [20]. In the first proposal for its routine use in clinical practice in 1869 [21], William Banting recognised even then the inevitable controversy and reputational risk it would evoke. In 1923, the “Dr. Elliot Joslin Diabetic Diet” consisted of “meats, poultry, game, fish, clear soups, gelatin, eggs, butter, olive oil, coffee, tea” with approximately 5% of calories from carbohydrates, 20% from protein, and 75% from fat [22].

We sought to conduct a narrative review of the role of low-carbohydrate diets for treating obesity and type 2 diabetes, to explore some of the controversies and to identify potential areas for clinical research prioritization and provide a better evidence base for patient care. In so doing we have endeavoured to put aside our personal “preferences” and biases and instead conduct a dispassionate, objective overview of the various issues giving rise to controversy and confusion.

## 2. What Is a Low Carbohydrate Diet?

The continuum of the degree of carbohydrate restriction that exists in contemporary clinical practice is illustrated in Table 1 [23], with general agreement that less than 20 g per day is a “very low” carbohydrate intake (though some use a threshold of less than 50 g), equivalent to about 10% of total energy intake. The threshold for “low” carbohydrate intake is usually accepted as less than 130 g/day, equivalent to less than 26% of total energy from carbohydrates. Consumption greater than 230 g per day is consistent with no restriction of carbohydrate, although we find that many of our patients with diabetes or obesity exceed several times this amount on a daily basis. In general, the greater the degree of carbohydrate restriction, the greater the degree of ketogenesis, such that carbohydrate intakes of more than 50 g per day are not usually sufficient for ketogenesis [10]. Hence, “low-carbohydrate” and “ketogenic” are not synonymous dietary terms, but do overlap.

International consensus guidelines on the dietary management of type 2 diabetes now endorse carbohydrate restriction as a legitimate therapeutic strategy [19,24,25]. Clinical trials lasting up to two years have shown that low-carbohydrate diets (in which total and saturated fats have replaced carbohydrates) have had beneficial effects on excess body weight, lipids (including high-density lipoprotein (HDL) cholesterol and triglycerides, but not low-density lipoprotein LDL cholesterol) and glucose metabolism [26,27,28]. These findings come at a time when pervading nutritional epidemiological dogma around the harms of dietary fat is starting to change. As an expert panel commissioned by the British Medical Journal to address this uncertainty and controversy recently put it, “Despite decades of dietary advice that the lower the total fat content, the healthier the diet, researchers and public health authorities now agree that to consider the effect of total fat intake alone on health is meaningless [and that] different types of fats must be considered” [29].

## 3. Evolving Observational Evidence

A recent large multinational observational study (PURE—Prospective Urban Rural Epidemiology) of more than 135,000 individuals from 18 countries provided strong evidence for the health benefits of dietary fats and other macronutrients [30]. Somewhat unexpectedly, higher intakes both of total fat but also of saturated, monounsaturated, and polyunsaturated fats were associated with lower all-cause mortality, but not cardiovascular mortality. Moreover, there was an inverse association between saturated fat consumption and stroke incidence. Remarkably, higher carbohydrate intake was associated with increased total mortality. Although the usual caveats relating to causal inference (association rather than causation) from observational studies apply, these findings add to concerns that dietary guidelines which focus on limiting the intake of total and saturated fats, not to mention encouraging carbohydrate intake, may do more harm than good. However, as with many nutritional epidemiological studies, the PURE study was subject to criticism for reasons such as imprecise quantification of dietary intake, estimated sodium intake, and residual socioeconomic confounding [31,32]. Moreover, the findings were unexpected because they appeared to contradict other observational studies, such as an apparent 20% increased mortality in those consuming a low carbohydrate diet, according to a large recent meta-analysis by Seidelmann et al. [33], and increased cardiovascular, cancer and all-cause mortality associated with a lower carbohydrate intake in Greek adults [34]. A critically important limitation of all these studies, at least in terms of their relevance and generalizability to considerations of the safety and efficacy of carbohydrate restriction, is that the “low” carbohydrate groups still consumed 25% and up to 40% of their total energy intake from carbohydrates, well above the typical thresholds considered for carbohydrate restriction. Whether utilizing therapeutic carbohydrate restriction or not, all clinicians caring for patients with obesity and type 2 diabetes ought to be aware of the limitations of applying findings from large observational population-based studies, where even relatively low carbohydrate intake was well above that used clinically in low-carbohydrate interventions.

Recently, the methodological flaws and potential biases in Ancel Keys’ “Seven Countries Study” have been described [35]. This work impacted on dietary strategies to reduce cardiovascular risk for decades, demonizing dietary fats and leading to the “diet-heart hypothesis” [36]. It is important to concede, however, that some more recent and methodologically rigorous epidemiological studies have further supported Keys’ hypothesis. For example, in one study patients with newly diagnosed diabetes who adhere to current “low fat” dietary guidelines had better outcomes than patients who don’t adhere to the guidelines [37]. Moreover, a very large and robust prospective cohort study of US healthcare workers suggested that low-carbohydrate intake was associated with “a modest increase in overall mortality” of 12%, comparing extreme deciles, but without a statistically significant trend across the overall population [38]. So, while some of the older epidemiological observations were prone to bias and weak design, clinicians advocating carbohydrate restriction in their patients must be aware that large, contemporaneous, well-conducted, rigorous, and objective studies do not necessarily universally support this approach.

But even the largest, best-conducted observational studies in nutritional epidemiology have several limitations that have been well described previously [39,40] and are beyond the scope of this paper. Framed another way, the study above [38] tells us that American doctors and nurses who ate fewer carbohydrates in the 1980s and 1990s had higher mortality rates than those who ate more carbohydrates. However, it is at least plausible that health care professionals who weren’t adhering to healthy eating advice at the time were less inclined to have healthy behaviors in other domains, only some of which (e.g., smoking) could be quantified. The extent to which unmeasured and residual confounding affected these observational findings, so that what we see is “association not causation” is difficult to say and is in fact a generic and substantial problem in these types of studies.

Getting back to carbohydrates, it has been established for some time that different types of carbohydrate have varying effects on metabolism and health [41]. For example, fructose exerts particularly deleterious effects on the liver, driving fat accumulation [42]. While consideration is often made of “refined” (sugary) versus “unrefined” (starchy) carbohydrates in population-based studies, precise quantification of macronutrient subgroup composition is often impossible with self-reported diet recall methods. In considering the relevance of these observational nutritional epidemiological studies to clinical practice, we would contest that they should certainly not constitute grounds for dismissal of the validity or utilization of low-carbohydrate diets. Indeed, a comprehensive synthesis of the evidence supporting low-carbohydrate diets by Feinman et al. [23] identified twelve distinct points of evidence supporting carbohydrate restriction as a first-line therapy for type 2 diabetes. The authors eloquently argue how the epidemiologic measurements are supported by biochemical mechanisms and that the benefits of dietary carbohydrate restriction do not always require weight loss, something the authors of this paper have seen in their own clinical practice.

## 4. “A Calorie Is a Calorie”, and Hunger

Contrasting with the complexities and nuances of nutritional epidemiological discourse, the reductionist view that “a calorie is a calorie” and that overall energy balance is what determines body weight appeals to many scientists, clinicians and the general public alike. However, perhaps this is overly simplistic. It is of course true that weight gain is a manifestation of a relative excess of calories ingested compared to those expended. Indeed, this has been the basis for “isocaloric substitution” of different macronutrients in observational modeling studies and mechanistic dietary interventions [43,44], in order that modelled differences in body weight and related health outcomes from differing levels of macronutrients don’t arise from differences in calories consumed. However the fallibility of this “energy in, energy out” adage has been highlighted by overfeeding studies [45], where considerable inter-individual heterogeneity in the magnitude of weight gain is found in those exposed to similar relative caloric excess, reflecting variable activation of compensatory adaptive mechanisms.

What if the type of food ingested affects appetite and so also “calories in”? Put another way, what if the calories acquired from eating, for example, potatoes (which have a higher glycemic index [46] and act in an equivalent way to glucose gels used by elite athletes [47], but which feature prominently in healthy eating guidelines) produce a different physiological effect on dietary behaviors than, say, the same amount of calories from an avocado? If an individual is hungrier in the hours after the former compared to the latter, then the isocaloric substitution approach may nullify potentially important differences between the effects on appetite, satiety, and eating behavior of different macronutrients. Anecdotally, many of our patients often report reduced hunger and increased satiety after changing to a low-carbohydrate diet, something which has been studied in a randomized controlled trial (RCT) [48] in which the low-carb diet group was less bothered by hunger compared to the low-fat diet group, but which warrants further assessment in mechanistic studies and trials.

## 5. Limitations of Interventional Nutrition Research

Without understating the importance of RCT’s, which may often improve our understanding of the efficacy of dietary interventions, we should be cautious on relying on RCTs or meta-analysis alone. One of authors (T.K.) is a registered dietitian and has significant experience in using different dietary approaches for the management of type 2 diabetes and obesity with patients. While there are further controversies, which are outside the scope of this paper, as to whether nutritional epidemiology and RCTs are in fact “gold standard” for nutrition research, one cannot fail to mention the importance of patient choice and preference when counselling a patient through any dietary intervention.

Dietary intake is influenced by many environmental, social, cultural, and economic factors. Relying strictly on RCT evidence does not allow us to take into account the critical influences of these factors on personal choice. Which of us, if allocated to a diet we found unacceptable as part of an RCT, would stick to it for even a few months let alone the years needed to supply long-term evidence?

## 6. Putative Physiological Mechanisms

**Figure 1:**
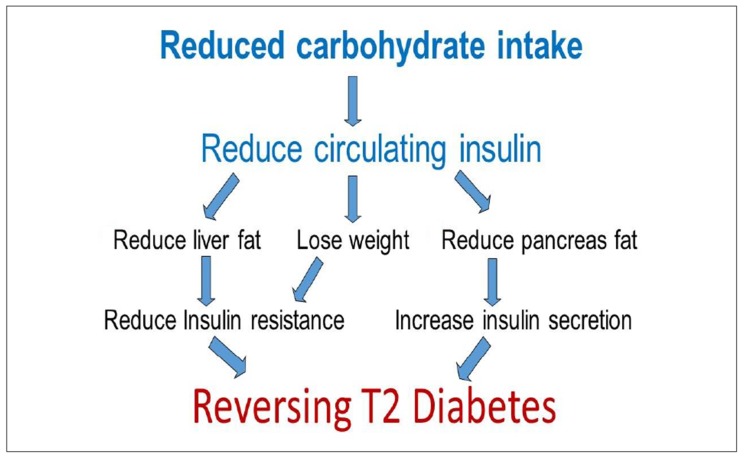
A simplistic model describing the mechanism by which reducing overall energy and carbohydrate intake may reverse the pathogenesis of type 2 diabetes. This model has been adapted with kind permission from Professor Roy Taylor, of the Diabetes Remission Clinical Trial (DiRECT) trial showing remission of type 2 diabetes in 46% of cases after one year when following a very low calorie diet.

It has been proposed that a shift from a hunter-gatherer “Paleolithic” diet in recent history has driven the epidemics of obesity, diabetes, and cardiovascular disease [49]. This would have had a relatively low proportion of carbohydrate, with (apart from honey) a low glycemic index [50]. By comparison, today’s western diets contain “dense acellular” carbohydrates with a higher glycemic index which could, among other things, promote an inflammatory microbiota leading to leptin resistance and obesity [51]. Twenty years ago, a study comparing subsistence horticulturalist native Papua New Guinea islanders with matched Northern European controls noted that their relative leanness and low burden of cardiovascular risk factors was associated with much lower insulin levels [52]. Carbohydrates have a much greater effect than fat or protein on insulin levels. High insulin levels are known to reduce satiety [53] and influence eating behavior [54] while down regulating lipolysis [55], Figure 1 depicts the mechanism by which carbohydrate reduction is thought to reduce insulin resistance [56]. A recent trial demonstrated that carbohydrate restriction is more beneficial for patients with higher insulin levels [44]. This is consistent with insulin [57,58] influencing the so-called “set point” [59] of eating behavior and body weight regulation, in the same way that efficacious interventions such as drug therapy [60] and surgery [61] do but which calorie restriction in isolation can’t [62]. Ketosis has been shown to attenuate the increases in ghrelin and appetite that occur with dietary restriction [63] and there is growing interest in the role of therapeutic ketosis in weight loss interventions [64].

The “carbohydrateinsulin model” predicts that diets with lower carbohydrate and higher fat (but identical calories) would reduce insulin secretion, leading to increased fat mobilization and oxidation [65]. Ultimately this would affect hunger and satiety, increasing body fat loss and energy expenditure compared with an isocaloric diet with higher carbohydrates and higher insulin secretion. This theory was recently refuted in a robust meta-analysis of 32 controlled feeding studies by Hall et al., where isocaloric substitution of carbohydrate for fat led to both reduced rather than increased energy expenditure and fat loss [66]. However, we would suggest that the same caveats as above should apply in discounting the effects of carbohydrates (as well as insulin or ketones) on dietary behavior and that the isocaloric substitution approach is potentially a misleading fallacy.

## 7. Clinical Effects of Nutritional Ketosis

The influence of nutritional ketosis from very low carbohydrate diets (VLCKDs) in adults with diabetes, obesity, and metabolic syndrome has been well described. Overweight adults with atherogenic dyslipidemia who were on such a diet for twelve weeks had favorable improvements in circulating free fatty acids and reductions in markers of vascular inflammation compared to those on an isocaloric low fat diet [67]. This amelioration in vascular inflammation has been shown to be mediated by beta-hydroxybutyrate (BOHB), inhibiting expression of the NLRP3 inflammasome [68]. BOHB also inhibits histone deacetylases, thus reducing oxidative stress [69]. Mechanistic studies in humans [70] and animals [71] suggest that infusion of BOHB improves left ventricular function in heart failure. While the effects of the diet on hepatic steatosis are thought to be both rapid and substantial [72], with animal studies [73] and post-hoc analyses in human trials [74] suggesting ketosis improves or prevents fatty liver, prospective randomized evidence is currently lacking. Similarly, reports of reductions in blood pressure and antihypertensive medication usage in observational studies and non-randomized trials are encouraging [75,76], but need to be assessed in prospective controlled trials. Conversely, there is good trial evidence that in patients with metabolic syndrome, adopting a low carbohydrate ketogenic diet is superior to other dietary strategies at improving lipid profiles [27,77].

Arguably the most significant and relevant therapeutic domain for nutritional ketosis is diabetes mellitus. In a recent small trial of patients with recently diagnosed type 2 diabetes, after 90 days of consuming less than 30 g of carbohydrate per day, mean weight loss was 9 kg, systolic and diastolic blood pressure reduced by 10.7 and 7.3 mmHg respectively, and mean Haemoglobin A1c (HbA1c) came down from 8.9% to 5.6% [78]. However, this study had just 11 patients and no control group, so while the validity of the findings is not in doubt, their generalizability and reproducibility remain to be determined. In a randomized controlled trial in overweight adults with an HbA1c above 6.0%, those on a VLCKD compared to moderate carbohydrate reduction lost more weight, had greater reductions in HbA1c, and stopped diabetes medication at twelve months [79]. A meta-analysis of nine studies with 734 patients suggested that low-carbohydrate (not necessarily ketogenic) diets in patients with type 2 diabetes led to 1.18 kg more weight loss and 0.44% greater reduction in HbA1c than normal- or high-carbohydrate diets [26]. A subsequent larger meta-analysis noted similar but less pronounced benefits across 18 studies, but also noted a high risk of bias, particularly performance bias in these studies, thus potentially diminishing internal validity [80]. However, lack of methodological rigor is a generic challenge in randomized controlled trials of dietary interventions. In a systematic review of 45 trials of dietary weight loss maintenance interventions in obese adults, only 10 had robust allocation concealment, 17 described some form of blinding, and 25 were deemed to handle incomplete data well. Others have confirmed that poor allocation concealment and blinding are particularly prevalent in these trials [81]. This creates potential for bias, residual confounding, and type 1 errors in some studies. Given the paucity of effective therapeutic nutritional strategies to reverse diabetes [82] and the potential promise shown by low carbohydrate and VLCKD diets [83], there is a need for their formal assessment in methodologically rigorous randomized controlled trials.

In the meantime, it is important not to dismiss the findings from other well-conducted studies, bearing in mind that inferences around efficacy, effectiveness, and safety need to be tempered according to study design. For example, several papers have described the effects of a commercial VLCKD programme, Virta^®^ Health, for patients with type 2 diabetes, using a cohort analysis of changes over time without a randomized control group. They have demonstrated rapid improvements in diabetes control within 70 days [84] with early down-titration of insulin and other diabetes medications [64] and with sustained improvements after two years [85]. These changes were associated with a reduction in cardiovascular risk [76] and fatty liver disease [74] and improved sleep [86] after a year. Weight loss of 12% was sustained after two years and retention was good [85]. On the face of it, providing patients with the opportunity to participate in such an intervention (in routine clinical practice, not just in academic research studies) seems appropriate and logical while we await higher-level evidence from randomized controlled trials.

Notwithstanding this, criticism of low-carbohydrate and ketogenic diets is widespread and pervasive, ever since early descriptions of their use by Jerome Conn, of eponymous hyperaldosteronism syndrome fame [87]. One recent commentary [88] noted negative results from some randomized controlled trials. However, the risk of type 2 statistical error may be significant. In one study, 60% of patients dropped out of the low-carbohydrate arm at two years, and the difference in weight loss between the groups of 0.2 versus 1.5 kg (favoring low-carbohydrate) may have been due to a lack of statistical power, not efficacy [89]. Furthermore, it is worrying that the supervising author was an investigator for a “competing” commercial weight loss program (Weightwatchers^®^), which wasn’t disclosed. Another “negative” low-carbohydrate trial had only 26 participants from a single center and while the duration of follow-up was two years, the low-carbohydrate intervention lasted only three months [90].

## 8. Clinical Practice Considerations

All of this controversy and uncertainty is reflected in the clinical practice of even the most qualified experts of all to provide therapeutic nutritional intervention—dietitians. A recent survey of their clinical practice in the UK found that only 48% of dietitians advised patients to restrict carbohydrate intake “occasionally or frequently”, with 35% of dietitians stating that they would consider between 30% and 39% of total energy intake from carbohydrates to be “realistic” [91]. Reflecting on our own extensive combined clinical experience with utilization of low-carbohydrate diets in patients with obesity or type 2 diabetes, we identified the following five points for consideration.

### 8.1. Guidelines Endorse Low-Carbohydrate and VLCKD Diets.

**Figure 2:**
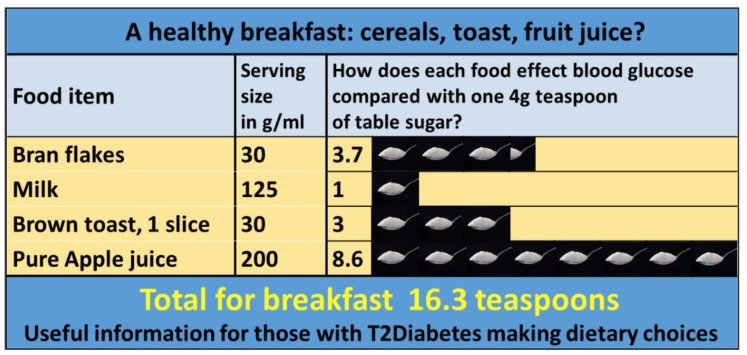
A NICE (National Institute of Clinical Excellence) endorsed infographic based on glycaemic load data, created by author D.U. showing the possible glycaemic consequence of a meal. Authors (T.K., F.F., D.U.) find these infographics extremely useful when explaining to patients the potential glycaemic consequences of a “healthy” breakfast. The authors acknowledge glycaemic response varies from individual to individual but find that a simple visual representation of the effect that certain foods have on blood glucose levels is helpful in supporting patients with their dietary choices.

Doctors, nurses and other health care professionals ought to be aware that the use of low-carbohydrate and VLCKD diets in patients with obesity or type 2 diabetes is in fact supported by several sets of guidelines from international bodies and professional groups, as outlined in Table 2. For example, the most recent guidance from the American Diabetes Association is unequivocal in stating that “Reducing overall carbohydrate intake for individuals with diabetes has demonstrated the most evidence for improving glycemia and may be applied in a variety of eating patterns that meet individual needs and preferences”. [86]. In the UK, pragmatic infographic resources based on the glycaemic load of various foods are available from the National Institute for Health and Care Excellence (NICE) (created by one of the authors (D.U.) [92]. See Figure 2 for an example. These help patients understand the glycemic “consequences” of their dietary choices. For example, a 150 g bowl of boiled rice has approximately an equivalent impact on blood glucose levels as ten standard teaspoons of table sugar.

### 8.2. Early De-Prescription Is Important

Although research data are relatively scarce on optimal patterns of medication usage early in low-carbohydrate and ketogenic diets, we have found that early and intensive de-prescribing is often required, particularly in patients with diabetes [93]. In particular, rapid titration of insulin is obviously important in order to prevent potentially serious hypoglycemia. (This clearly applies only to patients with an established diagnosis of insulin requiring type 2 diabetes, as opposed to type 1 diabetes: Low-carbohydrate diets have been shown to reduce adverse events and improve control in observational studies in patients with type 1 diabetes [94], but we have not considered this further here.) In general, we tend to stop all fast-acting insulin at the time of initiation of VLCKD and, if not stopping basal insulin completely, by then reducing the dose by between 50% and 80%. This mandates four-times-daily monitoring of capillary blood glucose levels in the hours and days after significant decreases in carbohydrate intake. We have found that it is essential that these patients have immediate access to a diabetes nurse, primary care doctor, consultant or dietitian with experience of low-carbohydrate diets during this time. In addition, we tend to stop sulphonylurea drugs completely at initiation of the diet because of the risk of hypoglycemia. Conversely, we tend to continue metformin given its insulin-sensitizing effects, cardiovascular benefits. and very low risk of hypoglycemia. We take an individualized approach to titrating gliptins or glitazones, informed by baseline HbA1c and patient preference. We often continue glucagon-like peptide-1 (GLP1) receptor agonists. Given the potential risk of euglycemic diabetic ketoacidosis [95] in patients taking sodium-glucose cotransporter-2 (SGLT2) inhibitor drugs, we always stop these if the diet is initiated.

The second group of drugs that need consideration during a low-carbohydrate diet is antihypertensive medications. This is because the higher circulating insulin levels in insulin resistant type 2 diabetes patients can cause renal sodium retention, which may be reversed quickly with a reduction in insulin levels (as part of a low-carbohydrate diet), leading to enhanced renal sodium (and water) excretion and a lower blood pressure [96]. We (D.U.) have described these changes in a cohort of 128 patients with type 2 diabetes on a low-carbohydrate diet for an average of two years [75], where there was a reduction in systolic and diastolic blood pressure of 10.9 and 6.3 mmHg, respectively, despite a 20% reduction in anti-hypertensive medication usage. The risk of hypotension mandates cautious and frequent monitoring of patients’ blood pressure and vigilance for symptoms of postural hypotension.

### 8.3. Calorie Counting Is Not Required

**Figure 3:**
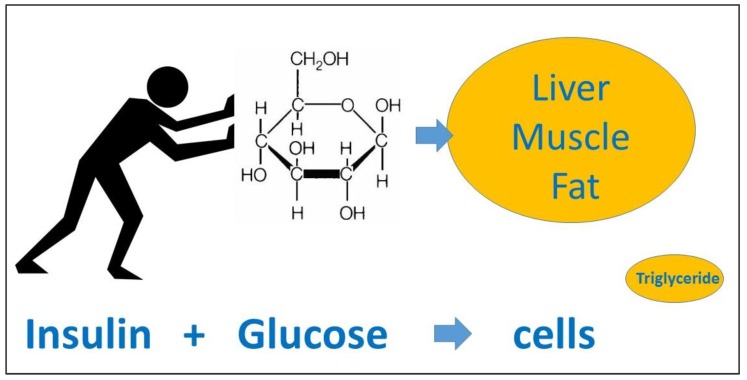
A useful model for explaining the physiology of Type 2 diabetes (T2D) to patients created by D.U. In clinical practice, the authors find it helpful to explain: Firstly that T2D is not just about dietary sugar as starch is “glucose molecules holding hands”. Digestion will break starch back down into glucose. Secondly, using the model shown here it helps to clarify how the hormone insulin “pushes” blood glucose into cells. Initially glucose is “pushed” into muscle cells for energy, however, if one consumes more sugar than is needed, the glucose starts being “pushed” into abdominal fat cells contributing to central obesity, and also into hepatocytes which metabolize the glucose into triglyceride, eventually potentially leading to fatty liver. Any resulting fatty liver itself can cause insulin resistance. For some people in a similar way, fat builds up in the pancreas interfering with beta-cell function and the production of insulin itself. This was described so well by Professor Taylor in his 2012 Banting lecture. It can be seen how this could contribute to the aetiology of T2D itself.

Rather than emphasizing the need for patients to quantify their calorie intake, we ask them to focus on eating to comfortable satiation and then stopping. The effectiveness of calorie counting has been questioned [97,98] with well-described physiological “recidivism” with this approach [99]. We take a more mechanistically intuitive approach, emphasizing to the patient that metabolic changes associated with their reduced carbohydrate intake may adjust their “hunger set-point” as outlined above. We often try to back this engagement and education up with graphical aids, emphasizing the physiological mechanisms underlying insulin resistance and their reversal with carbohydrate restriction, as shown in Figure 3. Our anecdotal experience of patients reporting significantly reduced hunger and increased satiety is consistent with studies on higher fat and protein diet influence on the physiological drivers of feeding behavior [100,101].

### 8.4. Monitor Cardiovascular Risk Factors

While all patients with obesity and type 2 diabetes should have cardiovascular risk factors monitored periodically, the potential increase in fat consumption that arises on a low-carbohydrate diet, in the context of historical epidemiological concerns that dietary fat might increase cardiovascular risk, makes the issue more pertinent. We have found that individuals concerned about the appropriateness of low-carbohydrate diets are either unfamiliar or don’t accept recent nutritional epidemiological discoveries around the ineffectiveness of low fat diets to prevent cardiovascular disease [29] and the benefits of low-carbohydrate intake [102] and certain saturated fats, such as those from dairy, in reducing cardiovascular (and diabetes) risk [103,104]. A recent meta-analysis suggested low-carbohydrate diets are superior to low fat diets in improving the lipid profile [105]. While current guidelines on saturated fat are overdue a revision [106,107], it seems reasonable to reassure patients undertaking a low-carbohydrate diet, and their health care providers, that saturated fats from foods that are not ultra-processed are unlikely to do them harm, especially if they are losing weight and improving glycaemia while undertaking a low-carbohydrate approach. A second consideration is the increase in LDL-cholesterol that is described with some [27] but not all [108] low-carbohydrate interventions, but the fact that this appears limited to the large LDL subfraction [76] suggests it is unclear whether it increases cardiovascular risk. Nonetheless, we routinely measure blood pressure, lipid profile and HbA1c in patients adhering to a low-carbohydrate or VLCKD and treat abnormal findings as we would in routine clinical practice, where they have not improved over time.

### 8.5. Ensure Adequate Fiber Intake

While the recommended intake of dietary fiber is 30 g per day (in the UK) the average intake is closer to 18 g per day. The reduction in wholegrain consumption associated with a low-carbohydrate diet could accentuate that deficit and low intake of dietary fiber is associated with an increased risk of metabolic [109] and colonic [110] disease. However, we have found in practice that adopting a low-carbohydrate diet which limits ultra-processed foods and includes nuts, seeds, non-starchy vegetables and low-carbohydrate fruits tends to lead to a net gain rather than a reduction in patients’ dietary fiber intake compared to baseline.

**Table 2:** Summary of current guidelines and consensus statements on the use of low-carbohydrate diets.

Body	Guideline	Year	Recommendation
Diabetes UK (UK)	Diabetes UK evidence-based nutrition guidelines for the prevention and management of diabetes	2011	The Diabetes UK 2011 guidelines support the view that low-carbohydrate diets may be considered an option for weight loss in people with Type 2 diabetes when supported by a registered healthcare professional. [111]
Scientific Advisory Committee on Nutrition (UK)	Carbohydrates and Health	2015	It is recommended that the dietary reference value for total carbohydrate should be maintained at an average population intake of approximately 50% of total dietary energy. [112]
SIGN Guidelines (UK)	Management of Diabetes—A National Clinical Guideline	2015	People with Type 2 Diabetes can be given dietary choices for achieving weight loss that may also improve glycaemic control. Options include simple calorie restriction, reducing fat intake, consumption of carbohydrates with a low rather than a high glycaemic index and restricting the total amount of dietary carbohydrate (a minimum of 50 g per day appears to be safe for up to 6 months). [113]
National Institute of Clinical Excellence (UK)	Type 2 diabetes in adults: management	2015	Individualise recommendations for carbohydrate and alcohol intake, and meal patterns.
American Diabetes Association and European Association for the Study of Diabetes (USA & Europe)	Management of Hyperglycaemia in Type 2 Diabetes. A consensus Report.	2018	**Nutritional therapies:** Low-carbohydrate, low-glycaemic index and high-protein diets, and the Dietary Approaches to Stop Hypertension (DASH) diet all improve glycaemic control, but the effect of the Mediterranean eating pattern appears to be the greatest. [25]
Diabetes Australia (Australia)	Low carbohydrate eating for people with diabetes—position statement	2018	For people with type 2 diabetes, there is reliable evidence that lower carb eating can be safe and useful in lowering average blood glucose levels in the short term (up to 6 months). It can also help reduce body weight and help manage heart disease risk factors such as raised cholesterol and raised blood pressure. All people with any type of diabetes who wish to follow a low carb diet should do so in consultation with their diabetes healthcare team. [114]
American Diabetes Association (USA)	Nutrition Therapy for Adults with Diabetes or Prediabetes: A Consensus Report	2019	Reducing overall carbohydrate intake for individuals with diabetes has demonstrated the most evidence for improving glycaemia and may be applied in a variety of eating patterns that meet individual needs and preferences. For select adults with type 2 diabetes not meeting glycaemic targets or where reducing anti-glycaemic medications is a priority, reducing overall carbohydrate intake with low- or very low-carbohydrate eating plans is a viable approach. [24]

## 9. Conclusions

Current guidelines support the use of low-carbohydrate diets as an alternative to standard low-fat, calorie-counting advice for suitable patients with obesity or type 2 diabetes. In patients on multiple medications and/or insulin or who have multiple dietary restrictions or other co-morbidities such as renal disease, low-carbohydrate diets should be supported by experienced health care professionals and a specialist dietitian who can facilitate optimal nutritional intake. Clinicians should continue with regular surveillance and management of other risk factors, even if diabetes remission is achieved [110]. The need for large multicenter randomized controlled trials with cardiovascular and other hard endpoints, as well as feasibility and cost-effectiveness analyses, seems warranted and worthy of prioritization. In particular it should be noted that, as with any other dietary interventions, the evidence for long-term compliance with, and sustainability of, carbohydrate restriction is currently not strong. In the absence of this higher-level evidence, existing data suggest that adopting a low-carbohydrate diet is a legitimate and potentially very effective treatment option for patients with diabetes and obesity.

## Figures and Tables

**Table 1:** Suggested definitions of different carbohydrate diets (Adapted from Feinman et al.) [23].

Description	Grams Per Day	Energy from Carbohydrate (%)
Ketogenic diet (very low carbohydrate diet)	<20–50 g	<10
Low Carbohydrate	<130 g	<26
Moderate Carbohydrate	130–230 g	26–45
High Carbohydrate	>230 g	>45
